# 

**Published:** 1851-03

**Authors:** 


### INDEX.

Abnormal developement of Liver and Spleen, by Dr. D. Hutchinson, 419
Allaire on Colloid Degeneration of the lung, 197
Allison on the use of Tanniu, 247
Amer. Med. Ass., Transactions of for 1849, notice of, 65, 133
Amer. Med. Ass., proceeedings of, for 1850, notice of, 162
Amer. Med, Ass., Transactions of, for 1850, notice of, 482
Amer. Med. Ass. for 1851, 496, 89
Analysis of the Prairie Soil, 334
Annual circulars of Medical Colleges. 255
Asthma treated by Nitric Acid, 329
Atlee, Prof. W. L.'s Introductory Lecture, notice of, 461
Attending families by the year, 405
Bark and Allum Injections in Vaginal Discharges, 395
Bell on Baths, notice of, 228
Bennett on the Uterus, notice of, 328
Brady's case of two fœtuses of unequal size at the same abortion, 314
Brainard's case of Tracheotomy, 316
Brainard on Injections in Dropsical Affections, 107
Bronchocele treated by compression, 487
Brooks, case of poisoning by Eating a Gar, 437
Carpenter on use and abuse of Alcohol, notice of, 324
Carpenter's Physiology, notice of, 73
Cazeau's Midwifery, notice of, 320
Cerebellum, connection of, with sexual function, 390
Chicago Medical Society, 88
Chambers on Neuralgia Uteri, 216
Chloroform as a Therapeutical agent, by Dr. J. Smick, 409
Chloroform in Surgical Cases, by Dr. Haldeman, 201
Cholera in Knox Co., Ill, by Dr. J. W. Spalding, 439
Cholera in Jamiaca, 488
Clinical Lectures and cases, by Prof. Davis, 425
Collodiou in Erysipelas, 489
Cholera, Treatment of, by Dr. B. F. Mullen, 306
Cholera in Chicago, 264
Churchill on Diseases of Infants and Children, notice of, 75
Churchill's Essay's on Puerperal Fever, notice of, 323
Cod-Liver Oil in Tubercles 154
Coloboma Iridis, by Dr. A. Garwood, 217
Collodion in Mammary Inflammation, 231 by Prof. Evans,
Collodion in Mammary Inflammation, & Small Pox, by Dr. J. H. Murphy, 423
Collodiou in Small Pox, 397
Collodion in Chilblains, by Prof. Evans, 433
Colloid Degeneration of the Lungs, by Dr. Allaire, 197
Conjestive Fever, by Dr. Sheldon, 206
" " by Dr. E. S. Cooper 299
Copeland on Apoplexy, notice of, 349
Cornell on Epilepsy, 251
Corns, Radical cure of, 160
Costiveness, treated by Nitro-Muriatic Acid, by Dr. E. McArthur, 218
Cotton Seed in Intermittent Fever, 396
Crural Phlebitis, by Dr. Hutchinson, 339
Crawford's case of absence of Uterus, 308
Cutaneous Poison, 404
Cutter's Anatomy, &c., notice of, 354
Davis's History of Medical Education and Institutions, 3, 91, 181, 267, 501
Davis, Prof. on connection of cerebellum with the Sexual Functions 390
Davis' notice of Southern Med. Rep. 385
Demonstrative Midwifery 88
Dowler's Response &c., notice of, 141
Dowler on the Necropolis of N. O., notice of, 350
Drake's Treatise on the Interior Valley of N. America, notice of 79, 142
Dunglison's Therapeutics, notice of, 321
Dunglison's Physiology, notice of, 352
Eerble on Children, notice of, 353
Editorial Etiquette, 335
Electro Galvanism by Dr. Washburn, 288
Ergotine in External Hemorrhoides, 393
Erratum, 500
Evans' Obstetrical Extractor. 53
Evans on Collodion in Mammary Inflammation, 213
Evans on Treatment of Rubeola, 315
Evans on Collodion in Chilblains, 433
Eve, Prof. P. F.'s Card, 399
Flint, Prof. A.'s Card, 398
Fœtuses, two of unequal size at the same abortion, by B. F. Brady, 314
Free Medical Schools, 82, 258
Garwood's case of Coloboma Iridis, 217
Graduates in Rush Med. Col. 1849-50. 86
Green's Introductory Lec., notice of, 461
Griffith's Formulary, notice of, 140
Halderman on Chloroform 201
Hathwell's case of Malformation, 50
Hemorrhsides with Scirhus Rectum, by Dr. R. Willard, 43
Hemorrhoids Treated by Fusa, 80
Hæmatocele, Dy Dr. R. Hall, 420
Homœopathic Impertinence, 337
Hutchinson's case of abnormal developement of Liver and Spleen, 419

Herrick, Prof. W. B.'s Valedictory notice of, 496
Hooping Cough, new treatment of, 241
Hutchinson on Crural Phlebitis, 339
Hydrocephalus, Iodide of Potassium in, by Dr. V. M. Satterlee, 218
Illinois General Hospital of the Lake, 263
Illinois State Medical Convention, 84
Illinois State Medical Society, 176
Illinois General Hospital, 492
Inoculation in Rubeola, by Dr. J. E. McGirr, 434
Injections in Dropsical Affections, by Prof. Brainard, 107
Iodine in Snake Bite, 403
Jarvis on Insanity, notice of, 351
Latta, Dr. S. A., on Homœopathy in Cholera in Cincinnati, notice of, 76
Lescher on Milk Sickness, 124
Lescher on change of position of spleen, 309
Maclaise's Surgical Anatomy, notice of, 178, 229, 333
Malaria, by Dr. S. Thompson, 19
Malformation, singular case of, by Dr. C. A. Hathwell, 50
Matthews on the use of Coffee, 46
McArthur on Netro-Muriatic Acid in costiveness, 218
Mattson on Consumption, notice of, 462
Medical Gleanings in Naples, 401
Medical Society of the Upper Illinois, 225
Medical Society of Logan, Mason & Menard Counties, Illinois, 179
Medical Societies, proceedings of, 229
Medical Topography, by Dr Goff, 415
Meek's Valedictory, 491
Medical Societies, 494
Midwifery and Diseases of Women, 478
Milk Sickness, by Dr. J. J. Lescher, 124
Ministers and Physicians 241
Miscellaneous Medical Intelligence, 88, 180, 264, 338
Mitchell's Materia Medica, notice of, 497
Mullen on Treatment of Cholera, 306
National Convention for resiving the Pharmacopœia, 156
Nellegan on Eruptive Diseases, 243
Neuralgia of the Uterus, by Dr. E. D. Chambers, 216
New Adhesive Agent, 161, 331
New Medical Journals 499, 260, 336
New Medical School, 138
New York State Medical Society, notice of, transactions of, 141
New method of operating for Hernia, 485
Nitrate of Silver in Dysintery, 238
Northern Lancet, notice of, 90
Oatman on Poisoning by Stramonium 317
Obituary Notices, 90, 180, 266, 500
Observations on the use of Coffee by Dr. W. Mathews, 53
Operative Surgery, by Prof. Mott, 400
Our Journal, 407
Palmer's Review of Committee on Medical Sciences, 355
Quicksilver in California, 471
Ranking's Abstract, notices of 79, 462
Reeses' Formulary, notice of, 333
Report of Committee on Practical Medicine, notice of, 372
Report of a suit for Libel, notice of, 233
Re-Vaccination in Prussia, 404
Ricord on Venerial disease, notice of, 227
Salts of Morphia, 482
Satterlee on Hydrocephalus 218
Scabies cured by Inunction, 244
Scaitica cured by Chloroform, 252
Scarlatina, treatment of by Inunction, 473
Sheldon on Congestive Fever, 206
Singular Revenge, 402
Smith, Prof. J. M.'s Anniversary Discourse, notice of, 461
Southern Med. Reports, review of, by Prof. Davis, 451
Southern Medical Reports, Review of, 385
Spirit of the Medical Press, 146
Spirometer, by Prof. Jackson, 463
Spleen, change of position of, by Dr. J. J. Lescher, 309
Statistics of Medical Schools, 87
Tannate of Quinine, 81
Taylor's Medical Juris. notice of, 319
Tilden & Co's Extracts, 236
Thayer on Uterine Hemorrhage, 63
The Physician and Patient, notice of, 406
The Dentists. 334
Thompson on Malaria, 19
Transactions of Amer. Med. Ass., for 1850, notice of, 441
Tracheotomy for abscess at the root of the tongue, by Prof. Brainard, 316
Treatment of Intermittent Fever, by a single dose of Quinine 336
Tubal Fœtation, by Dr. C. C. Warner, 220
Twins Singularly Connected, 235
Ulcers, treatment of by Opium 80
Uterine Hemorrhage, by Dr. Thayer, 63
Uterus, Absence of, by Dr. G. S. Crawford, 308
Valvular Disease of the Heart, 237
Valedictory, by Dr. E. G. Meek, 491
Venerial Affections, abstract of, 480
Warner's case of Tubal Fœtation, 220
Washburn on Electro Galvanism, 288
Western Medical Society of Wis., 64, 224
Willard on Hemorrhoides, 43
Wisconsin State Medical Society, 222

### NOTICE.

Having been appointed Chairman of the Committee on Surgery, by the Illinois State Medical Society, I will thank all members of the profession in the State to give me reports of improvements or discoveries in anything that pertains to the subject, which shall be noticed in the Report. D. BRAINARD.

Chicago, March, 1st, 1850.

### RECEIPTS FOR THE JOURNAL FOR JAN. & FEBRUARY.

### ILLINOIS.

| | | |
|---|---|---|
| Drs. | R. McCormick, | $6,00 |
| | W. J. Duckwall, | 2,00 |
| | Alvin Ballow, | 2,00 |
| | Thomas Hall, | 5,00 |
| | P. A. Allaire, | 2,00 |
| | M. D. Darnell, | 2,00 |
| | D. Ransom, | 2,00 |
| | Wm. Dudley, | 2,00 |
| | G. H. Gorham, | 5,00 |
| | C. Van Brunt, | 2,00 |
| | Samuel Wiley, | 2,00 |
| | E. Sandford, | 4,00 |
| | S. G. Moore, | 2,00 |
| | A. M. Johnson, | 2,00 |
| | F. R. Payne, | 2,00 |
| | J. B. E. Albright, | 2,00 |
| | H. H. Littlefild, | 2,00 |
| | John B. Glass, | 2,00 |
| | S. O. Long, | 2,00 |
| | —— Comstock, | 2,00 |
| | J. G. Griffith | 6,00 |
| | J. Woodworth, | 2,0 |
| | —— Trowbridge, | 2,00 |
| | R. P. Lamb, | 2,00 |
| | W. D. Nelson, | 2,00 |
| Drs. | J. J. Beatty, | $2,0[illegible] |
| | W. S. Gray, | 2,00 |
| | J. P. Dimmitt, | 2,00 |
| | D. C. Brown, | 2,00 |
| | Wm. McKnight, | 1,00 |
| | W. T. Hutchinson, | 4,00 |
| | B. Woodward, | 5,00 |

### INDIANA.

| | | |
|---|---|---|
| Drs. | L. A. Johnson, | $2,00 |
| | C. Van Fossen, | 2,00 |
| | J. Gibbons, | 2,00 |
| | T. Welch, | 6,00 |
| | L. Jewett, | 3,00 |
| | O. Richmond, | 2,00 |
| | J. H. Constant, | 2,00 |
| | Wm. B. Smith, | 2,00 |
| | E. H. Sutton, | 4.00 |
| | John Spell, | 2,00 |
| | John Pritchett, | 4,00 |
| | John E. Lloyd, | 2.00 |
| | James M. Ely, | 2,00 |
| | J. W. McKinney, | 2,00 |
| | J. S. Baxter, | 2,00 |
| | Samuel Ross, | 2,00 |
| | W. Lockhart, | 2,0 |
| Dr. | Hiram Duncan, | $2,00 |

### WISCONSIN.

| | | |
|---|---|---|
| Drs. | J. L. Scofield, | $2,0[illegible] |
| | J. Prentice, | 2,00 |
| | J. B. Talcott, | 6,00 |
| | Robert Dunlap, | 2,00 |
| | C. S. Blanchard, | 6,00 |
| | J. H. Babcock, | 2,00 |
| | M. B. Goff, | 2,00 |
| | C. A. Mills, | 4,00 |
| | O. S. Maxson, | 2,00 |

### MICHIGAN.

| | | |
|---|---|---|
| Drs. | J. K. Finley, | $2,00 |
| | I. J. Babcock, | 2,00 |
| | Tompkins & Garwood, | 3,00 |
| | A. Murray, | 2,00 |

### IOWA.

| | | |
|---|---|---|
| Drs. | R. W. Hall, | $2,00 |
| | F. Brady, | 2,00 |

### OHIO.

| | | |
|---|---|---|
| Drs. | L. Gishwiller, | $2,00 |
| | Geo. Watt, | 2,00 |

### NOTICE TO SUBSCRIBERS.

The *Intelligencer* will be sent on the first of April to *all* of our subscribers. Subsequently it will be sent to *those only* who shall have paid for the Journal at the time of the issue of each number. We shall print no extra copies of the Intelligencer; therefore to secure full sets of it, payment must be made by the 20th of May next. If any of our subscribers should wish to discontinue, they must notify us before the May number of the Journal is sent out. No discontinuence is allowed except at the close of the volume.

These rules will be rigidly adhered to, and no deviation from them need be expected.

We would again remind our friends, that *post paid letters* are the only kind to be taken from the office.

